# Costunolide and Parthenolide Ameliorate MPP+ Induced Apoptosis in the Cellular Parkinson’s Disease Model

**DOI:** 10.3390/cells12070992

**Published:** 2023-03-24

**Authors:** Mehmet Enes Arslan, Hasan Türkez, Yasemin Sevim, Harun Selvitopi, Abdurrahim Kadi, Sena Öner, Adil Mardinoğlu

**Affiliations:** 1 Department of Molecular Biology and Genetics, Faculty of Science, Erzurum Technical University, 25100 Erzurum, Turkey; 2 Department of Medical Biology, Faculty of Medicine, Atatürk University, 25240 Erzurum, Turkey; 3 Department of Mathematics, Faculty of Science, Erzurum Technical University, 25100 Erzurum, Turkey; 4 Science for Life Laboratory, KTH-Royal Institute of Technology, SE-17121 Stockholm, Sweden; 5 Centre for Host-Microbiome Interactions, Faculty of Dentistry, Oral & Craniofacial Sciences, King’s College London, London SE1 9RT, UK

**Keywords:** Parkinson’s disease, terpenoids, antioxidants, acetylcholinesterase, apoptosis

## Abstract

Monoamine oxidase B (MAO-B) is an enzyme that metabolizes several chemicals, including dopamine. MAO-B inhibitors are used in the treatment of Parkinson’s Disease (PD), and the inhibition of this enzyme reduces dopamine turnover and oxidative stress. The absence of dopamine results in PD pathogenesis originating from decreased Acetylcholinesterase (AChE) activity and elevated oxidative stress. Here, we performed a molecular docking analysis for the potential use of costunolide and parthenolide terpenoids as potential MAO-B inhibitors in the treatment of PD. Neuroprotective properties of plant-originated costunolide and parthenolide terpenoids were investigated in a cellular PD model that was developed by using MPP^+^ toxicity. We investigated neuroprotection mechanisms through the analysis of oxidative stress parameters, acetylcholinesterase activity and apoptotic cell death ratios. Our results showed that 100 µg/mL and 50 µg/mL of costunolide, and 50 µg/mL of parthenolide applied to the cellular disease model ameliorated the cytotoxicity caused by MPP^+^ exposure. We found that acetylcholinesterase activity assays exhibited that terpenoids could ameliorate and restore the enzyme activity as in negative control levels. The oxidative stress parameter analyses revealed that terpenoid application could enhance antioxidant levels and decrease oxidative stress in the cultures. In conclusion, we reported that these two terpenoid molecules could be used in the development of efficient treatment strategies for PD patients.

## 1. Introduction

Parkinson’s disease (PD) is a neurodegenerative disease (NDD) characterized by motor symptoms such as bradykinesia, akinesia, tremor, rigidity, and postural instability, as well as non-motor symptoms including fatigue, increased daytime sleepiness, depression, and anxiety [1]. There is no effective treatment to prevent the progression of the disease or ameliorate PD symptoms completely, thus there is an urgent need to develop potential agents and chemicals that slow down or halt disease progression [2]. While there is no cure for this disease, there are medications available that can help manage the symptoms. These drugs work by increasing the levels of dopamine in the brain, which is a chemical that is critical for the control of movement. The most common medications used to manage the symptoms of Parkinson’s disease include levodopa, dopamine agonists, and MAO-B inhibitors. Levodopa is a medication that is converted into dopamine in the brain. This medication is often used in the early stages of Parkinson’s disease and can be very effective in reducing symptoms. However, over time the effectiveness of levodopa may decrease and side effects may occur [3,4]. Dopamine agonists are another type of medication used to manage Parkinson’s disease symptoms. These drugs act like dopamine in the brain and can help reduce symptoms such as tremors and stiffness. However, these drugs can also have side effects such as hallucinations and compulsive behaviors. MAO-B inhibitors are a third type of medication used to manage Parkinson’s disease symptoms. These drugs work by blocking the enzyme MAO-B, which breaks down dopamine in the brain. By blocking this enzyme, these medications can increase the levels of dopamine in the brain and reduce symptoms of Parkinson’s disease [5,6,7]. It is important to work closely with a healthcare provider to determine the best treatment plan for managing Parkinson’s disease symptoms. This may include a combination of medications and other therapies such as physical therapy, speech therapy, and occupational therapy. With proper management, people with Parkinson’s disease can lead full and active lives.

Drug discovery studies have been targeting different key biochemical and enzymatic processes to slow down PD progression or reverse PD symptoms. One of the most important factors in PD progression is the oxidative stress factor, which mainly occurs in glial cell activation and mitochondrial dysfunction, and these mechanisms have been targeted to treat PD patients [8,9]. MPP+ based PD models have been very popular disease models for both in vitro and in vivo studies. MPTP (1-methyl-4-phenyl-1,2,3,6-tetrahydropyridine) is a similar molecule to the Paraquat herbicide and has been shown to stimulate Parkinsonian toxicity specifically in dopaminergic neurons. MPTP has been shown to be metabolized by the MAO-B enzyme to the active compound MPP+ and to block the activity of NADH-ubiquinone oxidoreductase (mitochondrial complex I). Finally, results have been shown in mitochondrial dysfunction, which leads to oxidative stress, apoptotic cell deaths and energy deficient status [10,11,12,13,14]. Moreover, acetylcholinesterase (AChE) activity is an important element for PD. AChE activity is decreased during the PD progression, and different drug candidates have targeted its enzyme activity to treat PD patients [15,16]. However, the treatment of PD may require targeting of the multiple pathways that do not cause severe toxicity and side effects, since PD is a multifactorial disease. Recent studies indicated that plant sources may have great potential both in improving antioxidant features and in modulating the enzyme activity of important targets [17,18,19]. 

Terpenes constitute the largest and most common group of >2000 chemical compounds identified as plant secondary metabolites [20]. They are mainly obtained from plants, for example coniferous plants such as pinus, abies, juniperus, and picea [21]. Terpenes are a class of hydrocarbons and constitute the main component of natural products such as essential oil, resin, wax and rubber [22]. Terpenes, consisting of isoprene units, have the chemical formula of (C_5_H_8_)n. Isoprene (2-methyl 1,3 butadiene), which is the basic structure of the terpenes, is formed of short carbon units containing five carbons and two double bonds. It is known that these units are connected in the form of head–tail to form compounds in straight chains or rings, and the classification of terpenes is made by the number of chains consisting of isoprene units [23,24]. Terpenes have been shown to have antioxidant, antimutagenic, anti-Parkinsonian and anticarcinogen properties, and they are widely used in aromatherapy and phytotherapy [25,26,27,28]. Terpenes, whose different properties have been determined, have been used in different sectors such as food, cleaning, cosmetics, medicine, and pharmacy [29]. In addition, in recent years it has been recommended to take various terpenes of plant origin as supplements for improving health status [30,31].

**Figure 1:**
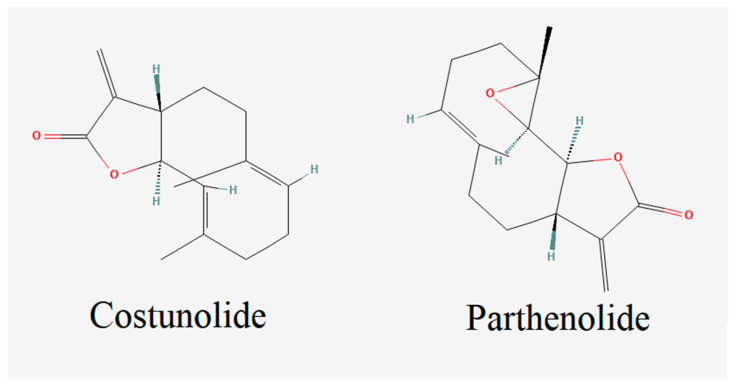
Chemical structures of costunolide and parthenolide terpenes.

Costunolide is a plant-derived terpene molecule that is extensively studied in different studies (Figure 1) [32]. Among the most appealing properties of costunolide are its antioxidant and anti-inflammatory features, which can be used against NDD. Various studies have claimed that costunolide decreases oxidative stress by increasing the glutathione (GSH) level increment and alleviating lipid peroxidation [33,34]. Moreover, parthenolide, which has antioxidant and anti-inflammatory properties, is another biologically active terpenoid compound that has great potential in the treatment of NDD [25,26,27]. A parthenolide derivative was shown to have a synergistic effect with L-DOPA against a 1-methyl-4-phenyl-1, 2, 3, 6-tetrahydropyridine (MPTP)-induced PD model in mice, and it was claimed that parthenolide administration can reduce L-DOPA doses in PD treatments [35]. Moreover, it has been reported that the parthenolide terpenoid enhances cell repair in spinal cord injury by regulating STAT 1/3 and NF-κB signaling pathways [36]. 

In the present study, we investigated anti-PD properties of costunolide and parthenolide terpenoids in differentiated SH-SY5Y cell cultures against MPP^+^ toxicity. SH-SY5Y cell lines were differentiated into mature neuron-like cell culture by sequential application of all trans-RA and BDNF. To enhance PD toxicity for the disease phenotype, the IC_50_ concentration of MPP+ compound was exposed to the differentiated cell line. Two different cell viability assays (MTT and LDH) were analyzed to determine the toxic doses of the compounds and neuroprotective concentrations of terpenoid molecules. We performed an AChE activity assay to determine the ameliorative effects of terpenoids on the enzyme activity. We also measured Total Antioxidant Status (TAS) and Total Oxidant Status (TOS) analyses to investigate the antioxidant potentials of terpenoids in PD cellular models. Hence, we investigated whether these plant-originated compounds have antioxidant and AChE activity alleviating effects and could be used in the treatment of PD patients. 

## 2. Materials and Methods

### 2.1. Molecular Docking

**Figure 2:**
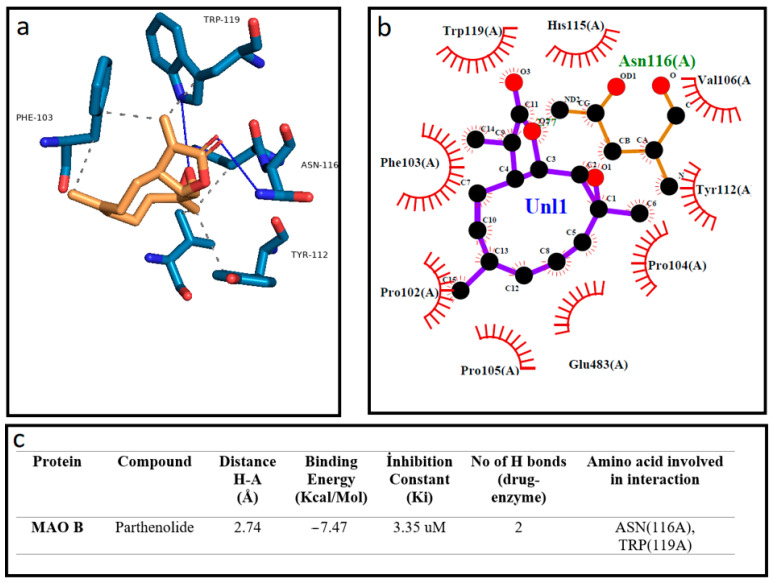
The best docking formation of the binding mode of Human Monoamine Oxidase B (Mao B-2BK5) with parthenolide obtained by (**a**)—PyMol, (**b**)—LigPlot, and (**c**)—the table of Hydrogen-bonds (H-bonds) (with distance Å), binding energy (Kcal/mol), inhibition constant, number of H-bonds (drug-enzyme) and amino acid involved in interaction obtained from the Protein-Ligand Interaction Profiler. The blue line and dashed gray show the H-bonds and Hydrophobic Interaction in Pymol, respectively.

Docking calculations were performed using Auto Dock software (AutoDock 4.2.6) downloaded from the Scripps Research Institute’s official website (http://autodock.scripps.edu/, accessed on 12 March 2022), with Phyton 3.10.5 and MGLTools 1.5.7. The software uses the genetic algorithm (GA) in the optimization step and runs 10 times in the GA processes, in accordance with the previously presented protocol [37]. The Human Monoamine Oxidase B (Mao B-2BK5) protein structure was downloaded from Protein Data Base (RCSB PDB, https://www.rcsb.org/, accessed on 11 January 2023) in the crystal structure. The structure of the molecules costunolide and parthenolide was acquired from the National Library of Medicine (PubChem, https://pubchem.ncbi.nlm.nsih.gov/, accessed on 11 January 2023). At the beginning of the docking process, water molecules were deleted and hydrogen atoms were added for the preparation of the protein in the AutoDock software. The obtained data from docking investigations from AutoDock software were analyzed via the use of Protein-Ligand Interaction Profiler (plip-tool.biotec.tu-dresden.de, accessed on 18 January 2023) online software, and the distance between ligand and amino acid residues were calculated in detail. PyMol software (pymol.org, accessed on 18 January 2023) was utilized to obtain 3D residual interaction plots. The LigPlot software (Version 4.5.3, https://www.ebi.ac.uk/thornton-srv/software/LIGPLOT, accessed on 18 January 2023) was used to obtain data related to interactions between the protein and ligand in 2D sketches [38,39]. Finally, we presented the comparison of the theoretical data obtained from the AutoDock software and experimental results. We observed that these results are highly in accordance with each other. The visualization of the binding affinities, H-bonds and other interactions between protein and ligand obtained via AutoDock 4.2.6 are presented in Figure 1 and Figure 2. 

### 2.2. Cell Culture and Neuronal Differentiation

A neuroblastoma (SH-SY5Y) cell line (ATCC, CRL-2266) was used to develop a cellular PD model for investigating the efficacy of terpenes on the disease phenotype. DMEM/F12 containing 5% fetal bovine serum and 1% penicillin/streptomycin was prepared for the cell culture growth in a 37 °C and 5% CO_2_ environment. After cell cultures covered the flask to about 80% density, 10 µM of all trans-retinoic acid (RA) in DMEM:F12 (2% fetal bovine serum) was administered to cultures and incubated for 7 days. Then, 50 ng/mL of BDNF was applied to the cell culture for another 3 days. Cellular differentiation was observed under an inverted microscope, and cell cycle analyses were performed to verify the differentiation through the use of a flow cytometer (The CyFlow^®^ Cube 6, Norderstedt, Germany) and real time PCR for tyrosine hydroxylase (TH), a DAergic neuronal marker.

### 2.3. Gene Expression Analyses of Differentiated Cell Cultures

Total RNA was isolated from the differentiated cell culture by using the PureLink RNA Mini Kit (Invitrogen, Grand Island, NY, USA) according to the manufacturer’s instructions. Briefly, 0.6 mL lysis buffer prepared with 2-mercaptoethanol was added to the cultures. Cell cultures were lysed via the use of 18–21-gauge needles by passing through 5–10 times. Cellular homogenates were washed with 70% ethanol and the sample was integrated into the spin cartridge and centrifuged at 12,000× *g* for 15 s at room temperature. Total RNA samples were eluted from the membrane by adding 100 μL of RNase–Free Water to the Spin Cartridge. Then, RNA quality was analyzed by using a plate reader (Multiskan, Thermo Labsystems, Vantaa, Finland) at a wavelength of 260 nm. Isolated RNA samples were reverse transcribed into cDNA by using a High-Capacity cDNA Reverse Transcription kit (Applied Biosystems, Foster City, CA, USA). Reverse Transcriptase, 10× RT Random Primers and nuclease-free H_2_O were used to prepare a reaction mix. RNA samples were added to the master mix, and reaction was performed in a thermal cycler (Sensoquest, Goettingen, Germany). The reaction was performed at 25 °C for 10 min, at 37 °C for 120 min, then at 85 °C for 5 min. For the real time PCR procedure, 102 μL cDNA samples, 1150 μL SYBR Green PCR Master Mix (Applied Biosystems), and 1048 μL RNase-free water was mixed. For each reaction, 2 μL of primer (Tyrosine hydroxylase (TH); Forward 5′-TGTGGCCTTTGAGGAGAAGGA and Reverse 5′-TCAAACACCTTCACAGCTCGG, beta-actin (ACTB); Forward→5′-TGACATCAAGGAGAAGCTCTGC-3′ and Reverse→5′-CCGCGGTGGCCATCT-3′, and glyceraldehyde-3-phosphate dehydrogenase (GAPDH); Forward→5′-GAGCACCAGGTGGTCTCC and Reverse→5′-TGAGCTTGACAAAGTGGTCG-3′) was mixed with 18 of PCR components for PCR reaction. Triple repeated reactions were applied for an initial denaturation step of 10 min at 95 °C, followed by 40 cycles of 15 s at 95 °C and 30 s at 60 °C. Data analysis was performed by the ΔΔCT method, with normalization of the raw data to 2 different housekeeping genes, including beta-actin (ACTB) and glyceraldehyde-3-phosphate dehydrogenase (GAPDH). Relative gene expression data were calculated using the Livak (2^−ΔΔCt^) method that normalized the CT values according to the reference genes. 

### 2.4. Cellular PD Toxicity Model Constitution

Differentiated SH-SY5Y cell cultures were seeded to 48-well plates in 10^5^ cell numbers to create a PD toxicity environment. Inhibitory doses of MPP^+^ were analyzed in a wide spectrum of concentrations (12–1500 μg/mL) for cell cultures. Then, 50% inhibitory concentration (IC_50_) of MPP+ was added into cell cultures to stimulate PD toxicity. IC_50_ value was calculated by using GraphPad Prism^®^ version 7.0 from Fit spline/LOWESS, interpolating unknowns from the standard curve option. Ameliorative effects of commercially available costunolide and parthenolide terpenes (Sigma-Aldrich^®^ Missouri, Germany) were analyzed in different concentrations (6.25–400 μg/mL) on the cellular PD model. After 24 h of incubation, triple biological repeated samples were analyzed using cytotoxicity assays. For the positive control, 10 µL of triton X (Sigma-Aldrich^®^ Germany) was applied to cell cultures at the end of incubation periods [40]. 

### 2.5. Cell Viability Analyses

For the cell viability analysis, lactate dehydrogenase (LDH) cytotoxicity (Cayman Chemical Company^®^, Ann Arbor, MI, USA) and 3-(4,5-Dimethylthiazol-2-yl)-2,5-diphenyltetrazolium bromide (MTT) cell viability assays were performed. Briefly, after the chemical treatment period ended (24 h), 100 µL of supernatants from cultures were transferred into fresh 48-well plates and 100 µL of LDH reaction mixture was added to the samples. Mixtures were incubated at room temperature for 30 min and color intensities were observed at 490 nm by using a microplate reader. An MTT assay kit (Sigma-Aldrich^®^, Burlington, MA, USA) was purchased to analyze the cell viabilities in cell cultures according to the manufacturer’s instructions. Then, 5 mg/mL of MTT solution was added to cell cultures and incubated at 37 °C for 3 h. Formazan crystals produced by health cells were dissolved by using an inorganic solvent (DMSO, Sigma-Aldrich^®^, Burlington, MA, USA). Finally, cell cultures were monitored by using a microplate reader at 570 nm wavelength.

### 2.6. Acetylcholinesterase (AChE) Activity Assay

Acetylcholinesterase (AChE) activity in the cellular PD model was analyzed by using a commercial kit (Abcam^®^, Cambridge, UK) according to the manufacturer’s instructions. Briefly, 24 h of chemically applied cell cultures were lysed via the use of the kit’s lysis buffer. Then, 50 μL of acetylthiocholine reaction buffer was added to each sample and incubated at room temperature for 30 min. Color intensities in the samples were monitored by using a microplate reader at 410 nm wavelength. To calculate the enzyme activities, absorbances in samples were compared to the kit’s standards.

### 2.7. Total Antioxidant Status (TAS) and Total Oxidant Status (TOS) Analyses

Total Antioxidant Status (TAS) and Total Oxidant Status (TOS) analyses were performed by using commercial kits (Rel Assay Diagnostics^®^, Gaziantep, Turkey). After 24 h of incubation with compounds, TAS analysis was performed by mixing 18 µL of cell culture supernatant (and kit’s standard-Trolox 1 mmol/L, and dH_2_O as positive and negative controls) with 300 µL Reagent 1 in 48-well plates. The first read was performed immediately after mixing the components at 660 nm absorbance. Then, samples were incubated at 37 °C for 5 min and measurements were performed again at 660 nm wavelength. The second measurement was subtracted from the first read and calculations were made according to the final results. TOS analysis was carried out by mixing 45 µL of the cell culture supernatant (and H_2_O_2_ 10 µmol/L and dH_2_O) and 300 µL of the kit’s reagent. Absorbance measurements were taken after mixing the samples at 530 nm. After that, kit reagent 2 was added to each sample, and mixtures were incubated at 37 °C for 5 min. Absorbance was monitored at 530 nm via the use of a microplate reader. 

### 2.8. Flow Cytometry Analyses

Apoptotic and necrotic status in cellular PD model against terpenoids treatment was determined by using an Annexin V-FITC Apoptosis Kit (Abcam^®^, BioVision, Cambridge, UK) according to the manufacturer’s instructions. Briefly, 10^5^ cells were seeded into a 48-well plate and compounds were applied to cell cultures for 24 h. Experimental analyses were divided into four groups: differentiated SH-SY5Y cell culture, MPP+ applied differentiated cell culture (cellular PD model), costunolide (100 µg/mL) applied cellular PD model and parthenolide (50 µg/mL) applied cellular PD model. Cell cultures were transferred into a fresh Eppendorf tube by using trypsin incubation for 3 min at 37 °C. Cells were centrifuged at 500× *g* for 5 min and supernatants were discarded. Cells were resuspended in 500 µL of Binding Buffer, and 5 µL of Annexin V-FITC/5 µL of propidium iodide was added to cell cultures. After 5 min of incubation at room temperature, cells were analyzed by flow cytometry (CyFlow^®^ Cube 6, Norderstedt, Germany).

### 2.9. Statistical Analysis

Statistical analyses for cytotoxicity and biochemical analysis were performed by using GraphPad Prism 7, ANOVA: Dunnett and Tukey comparison tests. Statistically significant parameters were calculated as *p* < 0.05.

## 3. Results

**Figure 3:**
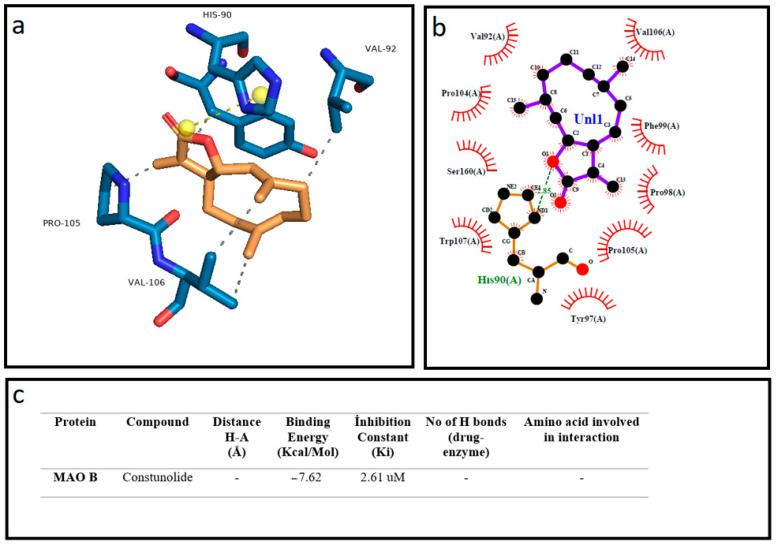
The best docking formation of the binding mode of Human Monoamine Oxidase B (Mao B-2BK5) with costunolide obtained by (**a**)—PyMol, (**b**)—LigPlot, and (**c**)—the table of Hydrogen-bonds (H-bonds) (with distance Å), binding energy (Kcal/mol), inhibition constant, number of H-bonds (drug-enzyme) and amino acid involved in interaction obtained from the Protein-Ligand Interaction Profiler. The blue line and dashed gray show the H-bonds and Hydrophobic Interaction in Pymol, respectively.

**Figure 4:**
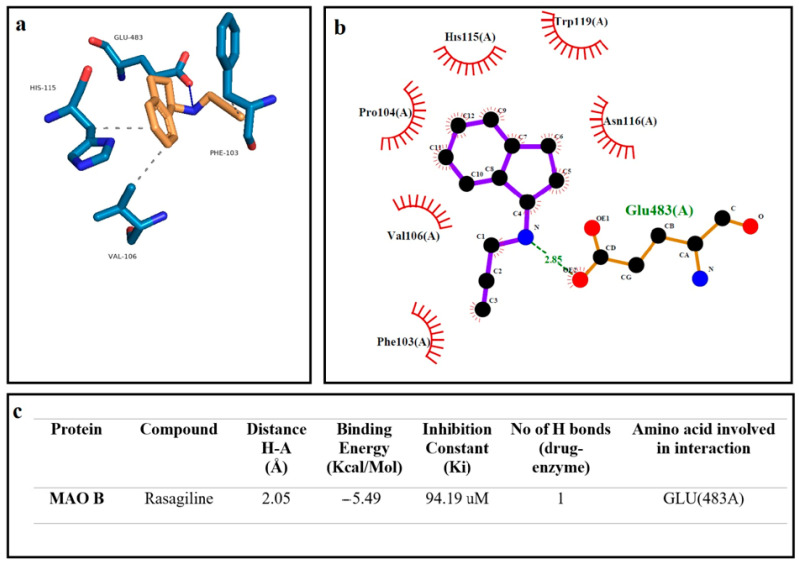
The best docking formation of the binding mode of Human Monoamine Oxidase B (Mao B-2BK5) with rasagiline obtained by (**a**)—PyMol, (**b**)—LigPlot, and (**c**)—the table of Hydrogen-bonds (H-bonds) (with distance Å), binding energy (Kcal/mol), inhibition constant, number of H-bonds (drug-enzyme) and amino acid involved in interaction obtained from the Protein-Ligand Interaction Profiler. The blue line and dashed gray show the H-bonds and Hydrophobic Interaction in Pymol, respectively.

We investigated the inhibitory effect of the terpenoid molecules on the MAO-B enzyme by using AutoDock software. Our analysis showed that costunolide and parthenolide have a higher affinity to MAO-B, with the lowest binding energies of −7.47 Kcal/Mol and −7.62 Kcal/Mol, respectively. Additionally, the parthenolide compound made two hydrogen bond interactions with MAO-B through ASN (116) and TRP (119) residues. Other hydrophobic interactions showed that costunolide and parthenolide have high affinity through several amino acid interactions (Figure 2 and Figure 3). On the other hand, a common anti-Parkinsonian drug, rasagiline, was analyzed by using a molecular docking approach to compare with the MAO-B inhibitory activity of candidate terpenoids. According to the investigations, rasagiline exhibited a lower binding affinity with a high binding energy of −5.49 Kcal/Mol compared to the terpenes (Figure 4).

**Figure 5:**
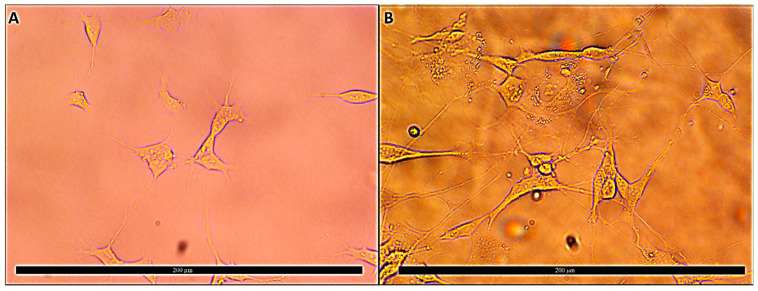
40x image of normal and differentiated SHSY-5Y cells. (**A**)—Undifferentiated SHSY-5Y cells and (**B**)—differentiated SHSY-5Y cells.

**Figure 6:**
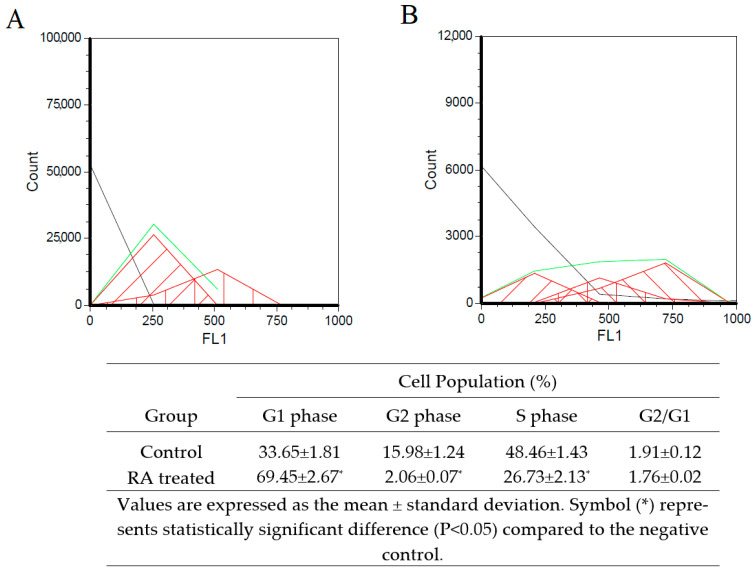
Cell cycle analysis of differentiated SH-SY5Y cells after 11 days of the retinoic acid application. (**A**)—Control (untreated SH-SY5Ycell culture) and (**B**)—RA treated cell culture.

**Figure 7:**
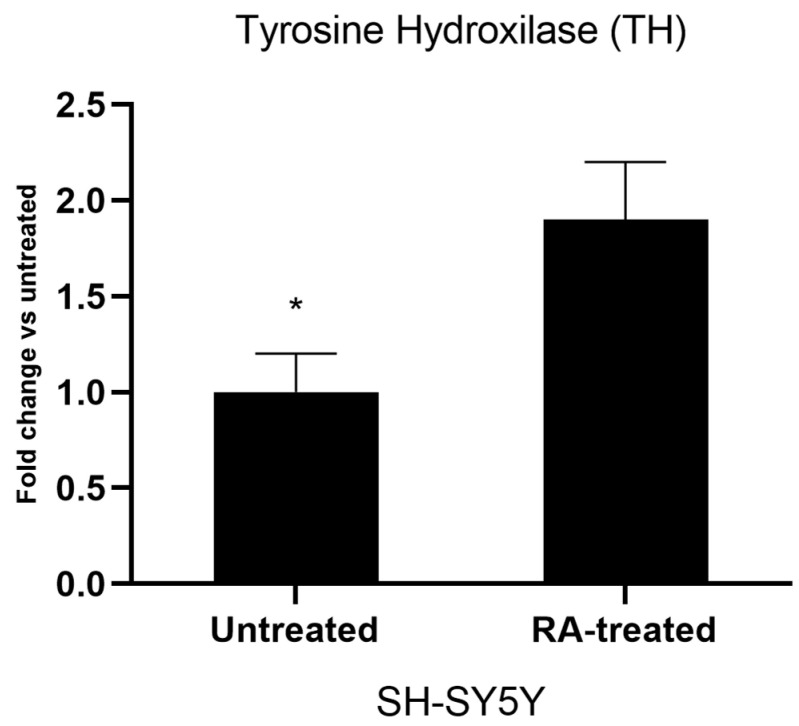
Gene expression analyses of untreated SH-SY5Y and RA-treated cell culture for tyrosine hydroxylase (TH). Statistical significance compared to untreated cell culture was shown as * *p* < 0.05. (GraphPad Prism^®^ version 7.0, Two-way ANOVA and Tukey’s post hoc test were used to compare the means of different treatments to determine significant difference (*p* < 0.05)).

The SH-SY5Y cell line was differentiated into a neuron-like cell culture via the serial application of all trans-RA and BDNF for 11 days. After the differentiation period, cell morphologies were switched from cubic-shaped structures into thinner body-like architectures. Axon and dendric-like compositions were assembled, and cell-to-cell attachments were visible to show cellular interactions under an inverted microscope (Figure 5). For further differentiation verification, cell cycle analyses of cell cultures were investigated by using flow cytometry. According to the results, cell populations in S phase (48.46 ± 1.43%) shifted into G1 phase (69.45 ± 2.67%). Additionally, G2 cell populations decreased from 15.98 ± 1.24% to 2.06 ± 0.07%. These results indicated that cell cultures may have decreased DNA synthesis and cell division, which could be claimed to be one of the characteristics of mature neurons (Figure 6). Moreover, the gene expression analysis of a DAergic neuronal marker, tyrosine hydroxylase (TH), put forth the suggestion that retinoic acid treatment for 11 days increased TH expression significantly compared to untreated SH-SY5Y cell cultures (Figure 7). 

**Figure 8:**
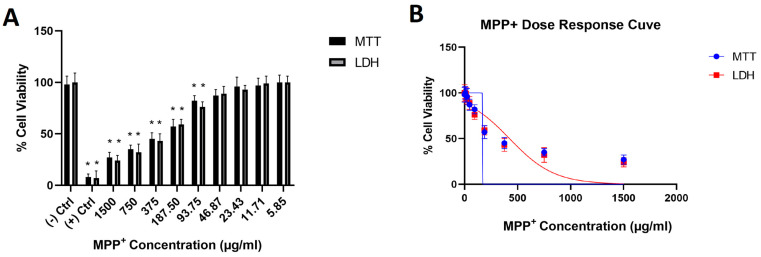
(**A**)—Cell viability of differentiated SHSY-5Y cells treated with MPP+ (24 h) is presented. (**B**)—Dose–response curve when the cell culture was treated with MPP+ in various concentrations. (−) Ctrl: Negative control. (+) Ctrl: Positive control (TritonX). Statistical significance compared to negative control was shown as * *p* < 0.05. (GraphPad Prism^®^ version 7.0, Two-way ANOVA and Tukey’s post hoc test were used to compare the means of different treatments to determine significant difference (*p* < 0.05)).

**Figure 9:**
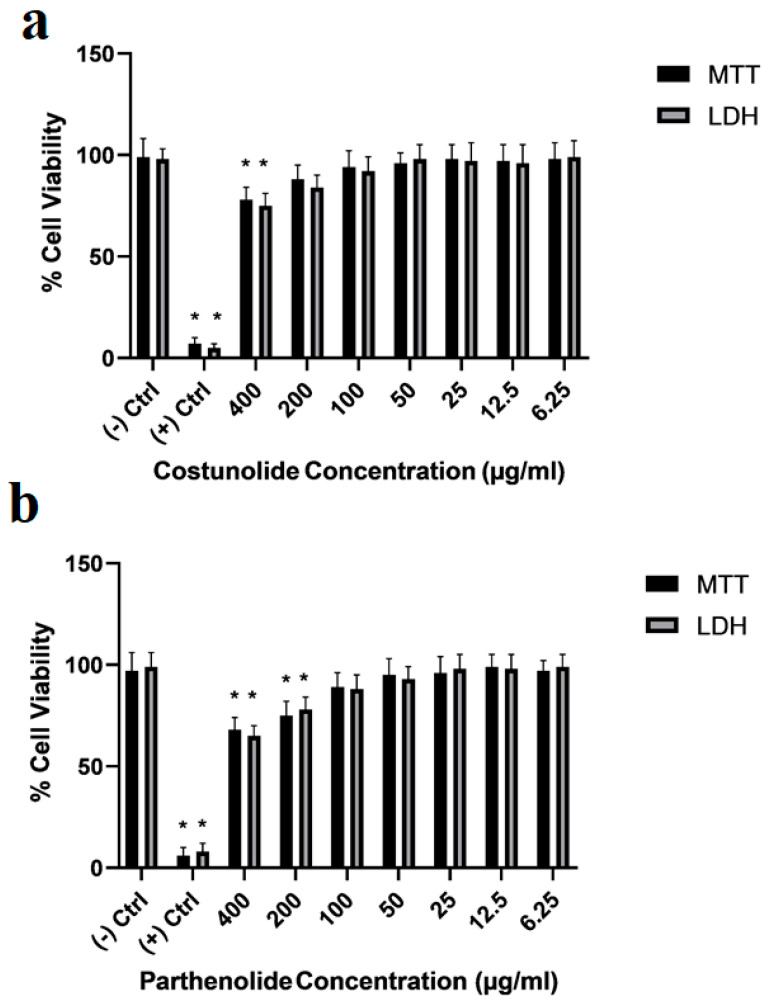
Viability rates of differentiated SHSY-5Y cells treated with (**a**)—costunolide and (**b**)—parthenolide terpenoids for 24 h are presented. (−) Ctrl: Negative control, (+) Ctrl: Positive control (Triton-X). Statistical significance compared to negative control was shown (*) *p* < 0.05. (GraphPad Prism^®^ version 7.0, Two-way ANOVA and Tukey’s post hoc test were used to compare the means of different treatments to determine significant difference (*p* < 0.05)).

To create a toxicological PD environment in differentiated SH-SY5Y cell cultures, a 50% inhibitory concentration (IC_50_) of MPP^+^ was determined by using MTT cell viability and LDH cytotoxicity assays. Our analyses showed that a 188 µg/mL concentration of MPP^+^ decreased 50% of the population of differentiated cell cultures (Figure 8). This concentration was used to analyze the neuroprotective properties of costunolide and parthenolide terpenoids in PD cellular models. Additionally, various concentrations of these terpenes were applied to differentiated SH-SY5Y cell cultures to determine non-toxic doses to treat PD models. Again, cell viability analyses were performed for both costunolide and parthenolide terpenes for 24 h in a wide range of concentrations. Our results showed that a 400 µg/mL concentration of costunolide, and 400 µg/mL and 200 µg/mL concentrations of parthenolide, significantly decreased differentiated cell numbers (Figure 9). 

**Figure 10:**
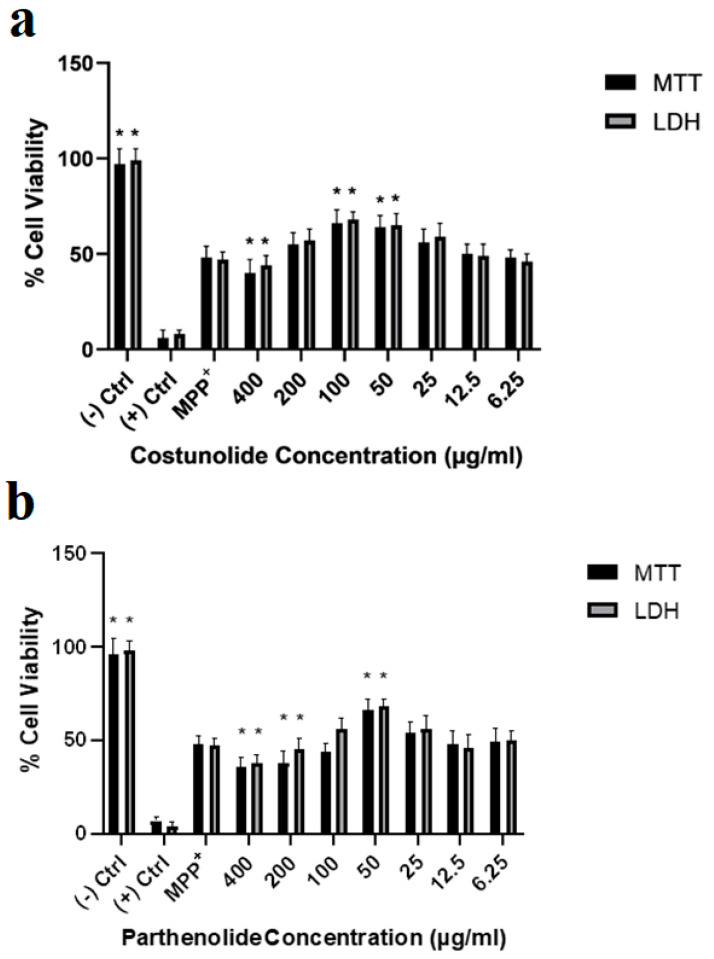
Cell viability analyses of SHSY-5Y cells treated with (**a**)—costunolide and (**b**)—parthenolide in an experimental in vitro Parkinson’s Disease model for 24 h. (−) Ctrl: Negative control, MPP+: MPP+ treated cells and (+) Ctrl: Positive control (Triton-X). Statistical significance (increase) compared to MPP+ control was shown as * *p* < 0.05. (GraphPad Prism^®^ version 7.0, Two-way ANOVA and Tukey’s post hoc test were used to compare the means of different treatments to determine significant difference (*p* < 0.05)).

Furthermore, PD toxicological cell culture models developed using SH-SY5Y differentiation and MPP^+^ (IC_50_, 188 µg/mL) treatment were investigated against costunolide and parthenolide molecules to test their neuroprotective properties. Cytotoxicity analyses showed that 100 µg/mL and 50 µg/mL of costunolide concentrations significantly increased the cell viabilities compared to only MPP+ applied groups in cellular PD models. On the other hand, 400 µg/mL of costunolide terpene decreased the cell viability compared to MPP+ applications (Figure 10a). Although parthenolide applications were shown to have cell viability enhancement ability in the 50 µg/mL concentration in PD cellular models, 400 µg/mL and 200 µg/mL of parthenolide exposures were investigated to significantly decrease the cell viabilities compared to only MPP^+^ applied groups (Figure 10b).

Acetylcholinesterase (AChE) activities were analyzed in the cellular PD model after the treatment with costunolide and parthenolide terpenes. Enzyme activity assay proposed that both costunolide and parthenolide applications did not change AChE activities in differentiated SH-SY5Y cell cultures compared to the negative control. On the other hand, MPP^+^ (188 µg/mL) exposure significantly increased the AChE activity (from 354.78 ± 9.18 mu/mL to 452.63 ± 12.42 mu/mL) in the differentiated cell cultures compared to the negative control. Additionally, when costunolide (100 µg/mL) and parthenolide (50 µg/mL) terpenoids were applied to cell cultures with MPP^+^ compound, we observed that terpenoids could ameliorate AChE inhibitory properties of MPP^+^ into its untreated conditions (from 452.63 ± 12.42 mu/mL to 365.45 ± 16.32 mu/mL and 357.35 ± 16.32 mu/mL, respectively) (Table 1).

Moreover, TAS and TOS assays were performed to analyze the antioxidant and oxidative stress conditions in the differentiated SH-SY5Y cell cultures with/without costunolide and parthenolide terpenes treatments against MPP+ toxicity. We found that MPP+ (188 µg/mL) exposure significantly decreased TAS levels from 1.86 ± 0.16 mM to 1.12 ± 0.05 mM (Trolox Equiv./L) compared to the negative control. However, costunolide (100 µg/mL) and parthenolide (50 µg/mL) treatments ameliorated the effect of MPP+ on TAS levels to 1.88 ± 0.21 mM and 1.76 ± 0.23 mM, which are similar values as in the negative control. In addition, we found that the TOS level was elevated from 5.54 ± 0.46 µM to 8.35 ± 0.89 µM (H_2_O_2_ Equiv./L) after MPP+ exposure in the differentiated cell cultures. In spite of that, after the treatment of costunolide (100 µg/mL) and parthenolide (50 µg/mL) into MPP+ (188 µg/mL) exposed cell cultures, the negative impact of MPP+ exposure was alleviated by decreasing TOS levels to 5.78 ± 0.52 µM and 5.85 ± 0.41 µM, respectively (Table 2).

**Figure 11:**
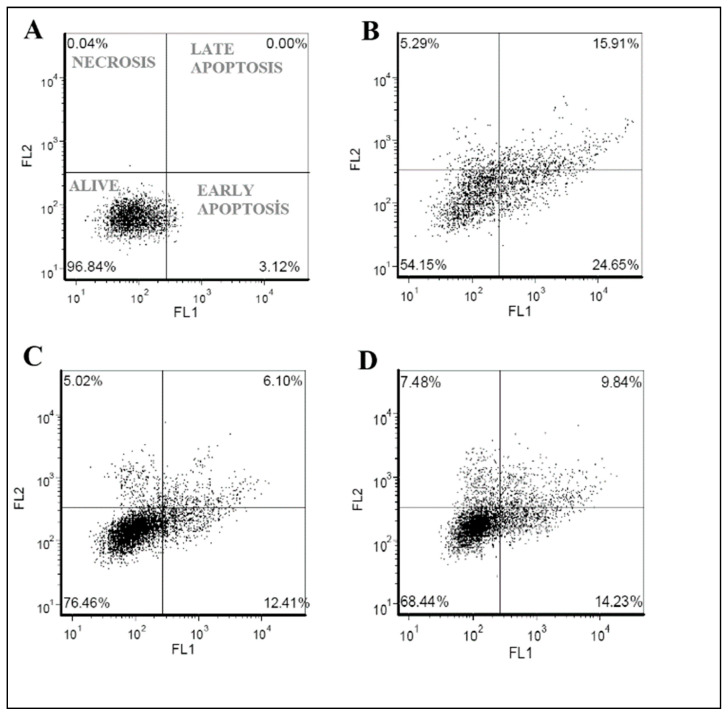
Flow cytometry analysis of compound application in cellular PD model. (**A**)—negative control (differentiated SH-SY5Y cell culture), (**B**)—MPP+ is applied to differentiated cell culture (cellular PD model), (**C**)—costunolide (100 µg/mL) is applied to cellular PD model, and (**D**)—parthenolide (50 µg/mL) is applied to cellular PD model.

**Figure 12:**
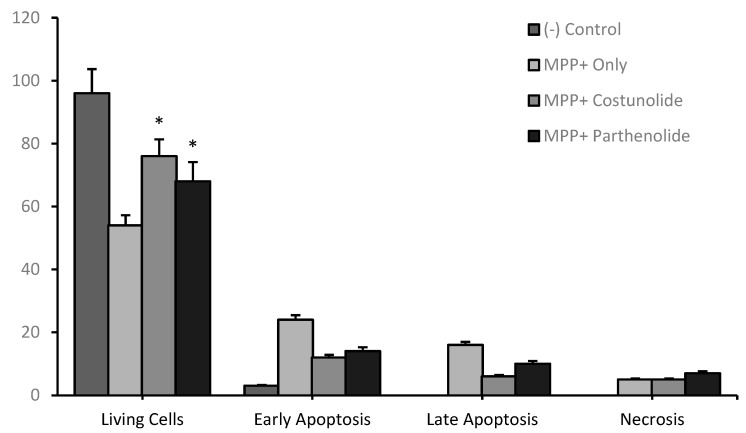
Flow cytometry analysis of costunolide and parthenolide applications (100 µg/mL) in the cellular PD model. Statistical significance compared to the MPP+ only applied group was shown (*) *p* < 0.05. (GraphPad Prism^®^ version 7.0, Two-way ANOVA and Tukey’s post hoc test were used to compare the means of different treatments to determine significant difference (*p* < 0.05)).

Finally, flow cytometry analyses were performed to investigate the cell death mechanisms in cellular PD models after terpenoid treatments. When MPP^+^ (188 µg/mL) was applied to the differentiated cell culture, 54.15% were alive and a total of 40.56% (early and late) apoptotic cell clusters were measured. On the other hand, when costunolide (100 µg/mL) terpenoid was applied to the cellular PD model, the alive cell ratio was increased to 76.46% and the apoptotic cell ratio was decreased to 18.51%. Similarly, after the application of parthenolide (50 µg/mL) to the cellular PD model the alive cell ratio was increased to 68.44 and the apoptotic cell ratio was decreased to 24.07% (Figure 11 and Figure 12).

## 4. Discussion

PD is one of the most aggressive dementia types, and symptoms of the disease affect patients’ lives quality severely. To date, there is no effective treatment to ameliorate the disease symptoms or cure the disease. Plant-originating metabolites and chemicals have been utilized to regulate multiple targets without causing adverse effects to other organs and systems [41,42,43]. In this study, two terpene family compounds, including costunolide and parthenolide, were tested to see if they could be used in ameliorating the PD toxicity by targeting antioxidant effect, AChE enzyme activity, and the apoptosis mechanism using MPP^+^ exposed differentiated SH-SY5Y cell cultures.

SH-SY5Y cell culture was differentiated by using all trans-RA to constitute neuron-like cell cultures for developing the PD phenotype in vitro. Different studies utilized this model to analyze various aspects of PD mechanisms, such as toxicological outcomes, genetic backgrounds, gene expression, and pathway regulations [44,45,46]. Differentiated cell cultures were investigated visually and by using flow cytometry cell cycle analysis. Morphologic changes such as thinner cell bodies, elongated dendrite, and axon structures in cell cultures indicated cellular differentiations. Additionally, cell cycle analysis confirmed that differentiated cells with decreased genomic DNA synthesis and increased cell ratio remained in the G1 phase.

Cell viability analyses were performed for the identification of IC_50_ concentrations of MPP^+^ application on differentiated SH-SY5Y cell cultures to stimulate the PD toxicologic environment. After the cellular PD model was created, different concentrations of costunolide and parthenolide terpenoids were applied to cultures to assess neuroprotective concentrations against MPP^+^ toxicity. Higher concentrations of costunolide (100 µg/mL and 50 µg/mL) were found to be effective in the cellular PD model compared to costunolide (50 µg/mL) treatments with regard to neuroprotection. On the other hand, the 400 µg/mL concentration of costunolide and 400 µg/mL and 200 µg/mL concentrations of parthenolide were found to show higher toxicity in MPP^+^ toxicity. The cell viability analyses of terpenoids indicate a significant toxicity in differentiated SH-SY5Y cell cultures without MPP^+^ exposure (Figure 5).

Effective doses of costunolide (100 µg/mL) and parthenolide (50 µg/mL) were applied to the cell cultures for biochemical analyses such as AChE activity assay, TAS/TOS investigations, and apoptosis/necrosis analysis. Previous studies claimed that different plant metabolites could inhibit AChE activity significantly in different NDD models [47,48,49]. Additionally, we showed that both costunolide and parthenolide applications to the MPP+ enhanced in vitro PD model decreased the AChE activity to the levels in the negative control. Ameliorated AChE activity resulted from the direct regulatory effects of costunolide and parthenolide treatments, such as preventive properties against MPP^+^/AChE regulatory features. Moreover, TAS and TOS assays indicated that terpenoids’ application increased the antioxidant levels and decreased the oxidative status in cellular PD models. It is well known that oxidative stress is an important factor for the pathogenesis of various diseases such as cancers, cardiovascular diseases and NDD [50,51,52]. Additionally, dopaminergic neurons have great vulnerability against oxidative stress, which can lead to PD phenotypes such as mitochondrial dysfunction, severe neuroinflammation, and higher apoptotic cell deaths [53]. Our results indicated that costunolide and parthenolide applications could enhance the antioxidant levels in cellular PD models to prevent various negative factors caused by MPP^+^ toxicological outcomes. Furthermore, our flow cytometry analyses confirmed the previous studies, where antioxidant applications to the MPP+ enhanced cellular PD models which can ameliorate apoptotic cell deaths and prevent dopaminergic neuron loss significantly were reported [54,55,56]. According to our study, 100 µg/mL of costunolide and 50 µg/mL of parthenolide applications to the in vitro PD model could reduce the apoptotic cell death ratio by 22% and 16.5%, respectively.

Moreover, we have developed the combined metabolic activators (CMAs), consisting of L-carnitine tartrate, GSH precursors (L-serine and N-Acetyl Cysteine) and NAD+ precursor (Nicotinamide or Nicotinamide Riboside), to increase the level of NAD+ as well as GSH levels [57,58]. We tested the effect of the CMAs in the animal models of NDD and found that CMA can be used in decreasing oxidative stress and activating mitochondria, and eventually reversing the disease-associated phenotypes in the animals [59]. We also tested the effect of the CMA in PD patients [58] and Alzheimer’s Disease patients [60], and found that the cognitive function of the AD and PD patients significantly improved in these two independent placebo-controlled studies. Hence, targeting antioxidant status, oxidative stress and mitochondrial activity for the treatment of NDD can be a strategy for the development of effective therapies for NDD patients.

## 5. Conclusions

In this study, we showed that costunolide and parthenolide terpenoids can be used to ameliorate the negative effects of MPP+ enhanced PD pathologies such as oxidative stress and apoptotic cell death. We also found that these molecules can be used in the inhibition of MAO-B enzyme activity. Terpenoids used in this study could also be used in targeting multiple mechanisms. The strong antioxidant properties of these plant-originating molecules also affect a specific cascade that triggers disease specific factors sequentially. These facts should be investigated in an isolated manner to find the exact target of costunolide and parthenolide terpenoids by running different in vitro and in vivo studies. Based on our findings, we concluded that costunolide and parthenolide can potentially be used for the development of efficient treatment strategies for PD patients after running animal and large clinical studies.

## Figures and Tables

**Table 1:** Acetylcholinesterase activity (mu/mL) of in vitro Parkinson’s Disease model with/without treatment of costunolide and parthenolide terpenoids after 24 h. One-way ANOVA, Dunnett tests were used for multiple comparison. Different letters in the same column present statistical significance from each value, *p* < 0.05.

Groups	Acetylcholinesterase Activity (mu/mL)
(−) Control	354.78 ± 9.18 ^a^
MPP+	452.63 ± 12.42 ^b^
Costunolide	335.75 ± 17.45 ^a^
Costunolide + MPP^+^	365.45 ± 16.32 ^a^
Parthenolide	325.28 ± 8.46 ^a^
Parthenolide + MPP^+^	357.35 ± 16.32 ^a^

**Table 2:** Total antioxidant status (TAS) and total oxidant status (TOS) levels in an experimental in vitro Parkinson’s model treated with costunolide and parthenolide terpenoids for 24 h. One-way ANOVA and Dunnett tests were used for multiple comparison. Different letters in the same column present statistical significance from each value, *p* < 0.05.

Groups	TAS (mM Trolox Equiv./L)	TOS (µM H_2_O_2_ Equiv./L)
(−) Control	1.86 ± 0.16 ^a^	5.54 ± 0.46 ^d^
MPP^+^	1.12 ± 0.05 ^b^	8.35 ± 0.89 ^e^
Costunolide	2.54 ± 0.15 ^c^	4.32 ± 0.33 ^f^
Costunolide + MPP^+^	1.88 ± 0.21 ^a^	5.78 ± 0.52 ^d^
Parthenolide	1.98 ± 0.22 ^a^	4.56 ± 0.63 ^f^
Parthenolide + MPP^+^	1.76 ± 0.23 ^a^	5.85 ± 0.41 ^d^

## Data Availability

The data presented in this study are available on request from the corresponding author. The data are not publicly available due to privacy.
